# Mortality associated with wildfire smoke exposure in Washington state, 2006–2017: a case-crossover study

**DOI:** 10.1186/s12940-020-0559-2

**Published:** 2020-01-13

**Authors:** Annie Doubleday, Jill Schulte, Lianne Sheppard, Matt Kadlec, Ranil Dhammapala, Julie Fox, Tania Busch Isaksen

**Affiliations:** 1 Department of Environmental and Occupational Health Sciences, University of Washington, 1959 NE Pacific St, Seattle, WA 98195 USA; 2 Air Quality Program, Washington State Department of Ecology, PO Box 47600, Olympia, WA 98504 USA; 3 Department of Biostatistics, University of Washington, 1705 NE Pacific St, Seattle, WA 98195 USA; 4 Office of Environmental Public Health Sciences, Washington State Department of Health, 243 Israel Road SE, Tumwater, WA 98501 USA

**Keywords:** Wildfire, Wildfire smoke, Environmental epidemiology, Mortality

## Abstract

**Background:**

Wildfire events are increasing in prevalence in the western United States. Research has found mixed results on the degree to which exposure to wildfire smoke is associated with an increased risk of mortality.

**Methods:**

We tested for an association between exposure to wildfire smoke and non-traumatic mortality in Washington State, USA. We characterized wildfire smoke days as binary for grid cells based on daily average PM_2.5_ concentrations, from June 1 through September 30, 2006–2017. Wildfire smoke days were defined as all days with assigned monitor concentration above a PM_2.5_ value of 20.4 μg/m^3^, with an additional set of criteria applied to days between 9 and 20.4 μg/m^3^. We employed a case-crossover study design using conditional logistic regression and time-stratified referent sampling, controlling for humidex.

**Results:**

The odds of all-ages non-traumatic mortality with same-day exposure was 1.0% (95% CI: − 1.0 - 4.0%) greater on wildfire smoke days compared to non-wildfire smoke days, and the previous day’s exposure was associated with a 2.0% (95% CI: 0.0–5.0%) increase. When stratified by cause of mortality, odds of same-day respiratory mortality increased by 9.0% (95% CI: 0.0–18.0%), while the odds of same-day COPD mortality increased by 14.0% (95% CI: 2.0–26.0%). In subgroup analyses, we observed a 35.0% (95% CI: 9.0–67.0%) increase in the odds of same-day respiratory mortality for adults ages 45–64.

**Conclusions:**

This study suggests increased odds of mortality in the first few days following wildfire smoke exposure. It is the first to examine this relationship in Washington State and will help inform local and state risk communication efforts and decision-making during future wildfire smoke events.

## Background

Wildfires are increasing in the western United States during the summer and fall months, emphasizing the importance of understanding the health impacts of wildfire smoke exposure [1, 2]. It is estimated that the total forest fire area burned in the western U.S. nearly doubled during 1984–2015 compared to the area projected to have burned without climate change [3]. This trend is expected to worsen, with climate projections indicating wildfires in the western U.S. will increase in frequency and intensity [4, 5]. The Intergovernmental Panel on Climate Change (IPCC) estimates that climate change will increase the length of wildfire season in North America by 10–30% [6], which is expected to result in worsening air quality during wildfire season in the coming decades [7].

Wildfire smoke contains a wide range of compounds known to be harmful to human health, including fine particulate matter (PM_2.5_), acrolein, benzene, carbon monoxide, and polycyclic aromatic hydrocarbons [8, 9]. Exposure to these toxic compounds is of concern near the source, and extending several hundred to thousands of kilometers away [10–12]. While it has been shown that the toxic compounds from wildfire smoke travel long distances from the source, potentially exposing thousands of individuals, the health effects associated with wildfire smoke exposure are just beginning to be understood [13].

Exposure assessment is challenging, as there is no standard approach for defining what constitutes a wildfire smoke-affected day or period in the health effects literature. Common methods utilize area monitoring particulate matter (PM) measurements, satellite data, chemical transport models, or a combination of these approaches [14–17]. Differences in effect estimates across studies in part may be due to differences in their exposure assessment approaches, limiting useful comparison within the growing published literature of wildfire smoke epidemiology. More research to define wildfire smoke-affected time periods is needed to validate methods currently in use.

Using the above exposure assessment methods, evidence for an association between exposure to wildfire smoke and all-age mortality is mixed. Four of five studies reported small increases in wildfire smoke associated mortality among all ages, however, all confidence intervals included the null [18–21]. The fifth study reported positive odds ratios (ORs) on lag days 0, 2, 3, and 4, but not on lag day 1 [22]. A study in Finland reported a 0.8% (95% CI: −3.5 – 5.3%) increase in all-cause mortality per 10 μg/m^3^ increase in same-day PM_2.5_ concentration [19], while another study in Australia estimated that wildfire events were associated with a 2.0% (95% CI: −2.0 – 5.0%) increase in the odds of same-day non-accidental mortality [21]. Several studies also report estimates for respiratory and cardiovascular mortality, finding limited evidence for an association with respiratory mortality [18, 21, 23] and small increases in the risk of cardiovascular mortality [18, 21].

A subset of these studies report larger effect estimates in groups 65 years and older [17, 24–26]. Analitis et al. reported that the effect of respiratory mortality in Greece was greater in adults age 75 and over during large fires [24]. Further, Haikerwal et al. observed an increase in risk of cardiac arrests, especially in older adults in Australia, although not all resulted in death [17], and Nunes et al. reported that older adults in their study in Brazil had the strongest association between exposure to biomass burning and circulatory disease mortality [26]. However, there are few U.S.-based mortality studies, providing little evidence for U.S.-specific mortality associated with wildfire smoke exposure. Additionally, there are no published mortality studies in Washington State, necessitating further research on the association between wildfire smoke exposure and non-traumatic mortality in the region.

Only two efforts have examined the health effects of wildfire smoke in Washington State, the 13th most populous state in the U.S., with an estimated 7.5 million people in 2018 [27]. Both of these efforts focused on the wildfire season of 2012, which was concentrated in Central Washington, resulting in limited population exposure [15, 28], and both studies examined hospitalizations, emergency department (ED) visits, and outpatient visits. No studies have been conducted on the risk of mortality associated with wildfire smoke exposure in Washington. Our study examines the association between wildfire smoke exposure and the odds of non-traumatic mortality in Washington State over 12 years. We hypothesized a priori that we would find a same-day effect and an effect at a lag of one or more days, within respiratory and cardiovascular mortality, and among individuals age 65 and older.

## Methods

### Mortality data

We conducted this study using Washington State geo-coded mortality and exposure data for wildfire season, defined as June through September, for our study period, 2006 to 2017. Historically, wildfire smoke has been documented in Washington during these months, and is described in an interagency operating plan as “peak fire season” in the Pacific Northwest [29]. Washington geo-coded mortality data includes latitude and longitude of residence, underlying cause of death, date of death, and decedent age, sex, and race. We examined non-traumatic causes of death (ICD-10 codes: A01-V99), including cardiovascular (ICD-10 codes: I05-I52); respiratory (ICD-10 codes: J01-J99); and cerebrovascular causes (ICD-10 codes: I60–67). About 2.7% of total cases were excluded due to missing latitude and longitude data (see Additional file 1: Figure S1).

We obtained median household income data at the census tract level from the U.S. Census Bureau for 2010–2017 and extrapolated to 2006 based on the five-year average percent change in growth within each census tract [30]. For the median household income analysis only, we omitted 12.9% of cases due to missing or incomplete household income data (see Additional file 1: Figure S1).

### Exposure data

Common approaches to assessment of outdoor PM_2.5_ exposure include assigning subjects’ data from their nearest stationary monitor and a variety of modeling and interpolation techniques [31]. The “nearest monitor” approach is not adequate in Washington due to the complexity of Pacific Northwest meteorology and terrain [32]. Since accurate daily particulate matter concentrations were required, modeled or interpolated products alone could not provide sufficient daily accuracy for such an analysis, particularly during wildfire smoke conditions [33].

For the exposure assessment, we used 4 × 4 km grid cells from the *Air Indicator Report for Public Awareness and Community Tracking (AIRPACT-4)* model domain [34]. We assigned each grid cell in the domain to one of three regulatory air quality monitors closest to it, of the 75 regulatory air quality monitors in Washington (see Additional file 1: Figure S2), using an interpolated surface of summer mean PM_2.5_ concentrations in smoke-free conditions. This method matched grid cells to nearby monitors based on typical agreement between the interpolated PM_2.5_ at the grid cell and the monitor. Grid cells were assigned to a secondary or tertiary monitor on days when data at the primary monitor were not available, which decreased data loss due to missing exposure data (see Additional file 1: Text S1, for additional detail). We then assigned each grid cell the daily PM_2.5_ concentration from its assigned monitor on that day and meteorological variables from the monitor’s nearest National Weather Service meteorological site. With this approach, we could leverage the accuracy of daily measurement data while improving upon the typical approach of simply assigning the nearest monitor. The result is a dataset with the following for each day and each grid cell: 24-h average PM_2.5_ concentration and humidex, a measure of apparent temperature calculated from air temperature and dew point [35].

About 1.5% of person-days across the study time period were not considered to be represented by any monitoring site, and were excluded from the analysis (see Additional file 1: Figure S3, for a map showing areas excluded). This resulted in the exclusion of 1.2% of all non-traumatic deaths in Washington during the study period (see Additional file 1: Figure S1). We joined this dataset with the above described mortality data using a spatial join in ArcGIS (version 10.5.1; Esri, Redlands, CA), assigning the latitude and longitude of the residence of each decedent to the nearest grid cell and corresponding PM_2.5_ concentration and humidex value.

### Wildfire smoke day classification

In order to identify wildfire smoke-affected days, we considered a number of approaches. First, a statewide PM_2.5_ concentration was tested as the threshold between wildfire smoke and non-wildfire smoke days, set at 15 μg/m^3^, corresponding to the 99th percentile of measured PM_2,5_ concentrations across two relatively smoke free years. The majority of days that exceeded 15 μg/m^3^ were recorded in urban areas without wildfire smoke contributions. Thus, a more nuanced and area-specific approach was needed to minimize false positives in urban areas with higher background particulate matter and to minimize false negatives in rural areas with lower background particulate matter [36, 37]. We defined wildfire smoke-affected days as grid cell days with a 24-h average PM_2.5_ concentration greater than 20.4 μg/m^3^, with an additional set of criteria for days between 9 and 20.4 μg/m^3^. A concentration of 20.4 μg/m^3^ corresponds to the threshold between Moderate and Unhealthy for Sensitive Groups Air Quality by the Washington Air Quality Advisory [38], and we found background anthropogenic particulate matter across the study period to be below this level. For days with a 24-h average PM_2.5_ concentration between 9 and 20.4 μg/m3, we applied the following criteria:

1. The day must be part of an event in which at least 2 of 3 consecutive days are greater than 9 μg/m^3^;
2. One of the days in the 3-day event window must be greater than 15 μg/m^3^;
3. For urban areas (Seattle, Tacoma, Spokane), at least 50% of the air monitors in those areas must be greater than 9 μg/m^3^ (see Additional file 1: Text S2, for a definition of urban areas).

We selected these criteria due to the nature of wildfire smoke events in Washington State. The first two criteria were informed by the historical observation that nearly all smoke events span multiple days, and the third criterion was informed by the observation that smoke events tend to affect nearby monitors in a region.

### Statistical analysis

We employed a time-stratified case-crossover design to examine the association between wildfire smoke exposure and non-traumatic mortality, using conditional logistic regression. This study design compares wildfire smoke exposure, defined as the binary wildfire smoke day classification described above, on the day of death, the day prior to death, and on the 4 days prior to death, to wildfire smoke exposure on referent (non-event, or control) days for the same decedent. We selected referent days using time-stratified sampling, where we defined the strata as the same day of the week, month, and year of death, yielding 3.39 referent days per decedent, on average. By design, this technique controls for time-invariant confounders, including sex, age, race, pre-existing health conditions, and other individual characteristics and risk factors, as each person serves as their own control [39]. This design also controls for some time-dependent variables based on the referent selection method, including day of the week, and seasonal trends in air pollution [40]. We reported results as a percent change in the odds of non-traumatic mortality for wildfire smoke-affected days versus non-wildfire smoke-affected days, after controlling for humidex. We adjusted for humidex by adding a term into the conditional logistic regression estimating equation. We used the *clogit* function in the *survival* R package to conduct the regression analysis [41].

To examine the effect of wildfire smoke exposure by characteristics of interest, we conducted subgroup analyses, stratifying by sex, age group, race category, cause of death, location (urban and rural), and census tract median household income, stratified by income groups shown in Table 1, for both same-day and previous-day exposures. We also conducted a lag analysis using an unconstrained distributed lag model from days 0–4, with day 0 modeled as the day of death, lag day 1 as the previous day, and so on.

**Table 1:** Non-traumatic mortality characteristics

Characteristic	N (%)	N (%) with exposure variation^a^
Total	170,985 (100)	31,719
Age group (years)		
0–4	2279 (1.3)	422 (1.3)
5–14	661 (0.4)	139 (0.4)
15–44	4912 (2.9)	934 (3.0)
45–64	31,956 (18.7)	6062 (19.2)
65–84	74,200 (43.3)	13,678 (43.2)
85+	56,977 (33.3)	10,391 (32.9)
Death day of week		
Monday	24,165 (14.1)	4623 (14.6)
Tuesday	24,202 (14.2)	5035 (15.9)
Wednesday	24,292 (14.2)	4914 (15.5)
Thursday	24,507 (14.3)	4675 (14.8)
Friday	24,902 (14.6)	3858 (12.2)
Saturday	24,800 (14.5)	4378 (13.8)
Sunday	24,117 (14.1)	4143 (13.1)
Location		
Non-urban	86,359 (50.5)	13,856 (43.8)
Urban	84,626 (49.5)	17,770 (56.2)
Median household income^b^		
< $35,000	16,039 (9.4)	3663 (11.6)
$35,000 - $50,000	41,693 (24.4)	7901 (25.0)
$50,000 - $75,000	62,703 (36.7)	10,990 (34.7)
$75,000 - $100,000	24,549 (14.4)	4572 (14.5)
≥ $100,000	8156 (4.8)	1442 (4.6)
Not reported	17,845 (10.4)	3058 (9.7)
Race		
White	154,311 (90.2)	28,309 (89.5)
Black	4665 (2.7)	883 (2.8)
Native American	2429 (1.4)	471 (1.5)
Hispanic	2219 (1.3)	579 (1.8)
Native Hawaiian/ Other Pacific Islander	1620 (0.9)	329 (1.0)
Asian	5193 (3.0)	941 (3.0)
Not reported	548 (0.3)	114 (0.4)
Sex		
Female	86,479 (50.6)	15,893 (50.3)
Male	84,513 (49.4)	15,732 (49.7)
Not reported	2 (0)	1 (0)
Underlying cause of death		
Cardiovascular	44,372 (26.0)	8120 (25.7)
Ischemic heart disease	7912 (4.6)	1477 (4.7)
Respiratory	13,355 (7.8)	2933 (9.3)
Asthma	253 (0.1)	46 (0.1)
COPD	9528 (5.6)	1726 (5.5)
Pneumonia	2166 (1.3)	377 (1.2)
Cerebrovascular	3732 (2.2)	708 (2.2)

^a^Percent of cases, out of all individuals with an event in a referent window with exposure variation

^b^Annual median household income estimates at the census tract level

We conducted secondary subgroup analyses for same-day exposures. We reported estimates by age group and race within respiratory causes of death, and by age group for Chronic Obstructive Pulmonary Disease (COPD) causes of death (Table 4). We also conducted a sensitivity analysis, setting a PM_2.5_ concentration of 20.4 μg/m^3^ as the wildfire smoke-affected day threshold, without additional criteria for days between 9 and 20.4 μg/m^3^, to assess whether our results were sensitive to the exposure definition (see Additional file 1: Table S1).

All analyses were performed using R 3.4.3 [41].

## Results

Table 1 summarizes characteristics of the 170,985 non-traumatic deaths included in our study in Washington from June through September for 2006–2017. Most non-traumatic deaths occurred in those 65 years and older (76.6%), most were white race (90.2%), and most lived in census tracts with a median household income of less than $75,000 (70.5%). About a quarter of deaths were due to cardiovascular causes (26.0%) and less than 10% were due to respiratory causes (7.8%). Table 1 also reports the number and percent of deaths that contribute to the inferential analysis, defined as belonging to a stratum with exposure variation, i.e. containing both wildfire smoke and non-wildfire smoke days.

In Table 2, we display exposure characteristics across the study period, including mean PM_2.5_ and humidex on wildfire smoke days and non-wildfire smoke days, and average PM_2.5_ on event and referent days.

**Table 2:** Daily PM_2.5_ characteristics for mortality days and referent days

Characteristic		Number
Number of exposure grid cells		10,106
Average number of wildfire smoke days per grid cell per year		13.1 (SD: 10.8)
Characteristic		PM_2.5_ (μg/m^3^) Mean (SD)
Event days (day of death)		6.38 (9.28)
Referent days		6.35 (9.11)
Characteristic		% Wildfire smoke days
Event days (day of death)		5.78
Referent days		5.73
Exposure metric	PM_2.5_ (μg/m^3^) Mean (SD)	Humidex Mean (SD)
Wildfire Smoke days	26.4 (31.9)	29.9 (5.53)
Non-Wildfire Smoke days	4.67 (2.53)	24.9 (6.03)

**Fig. 1:**
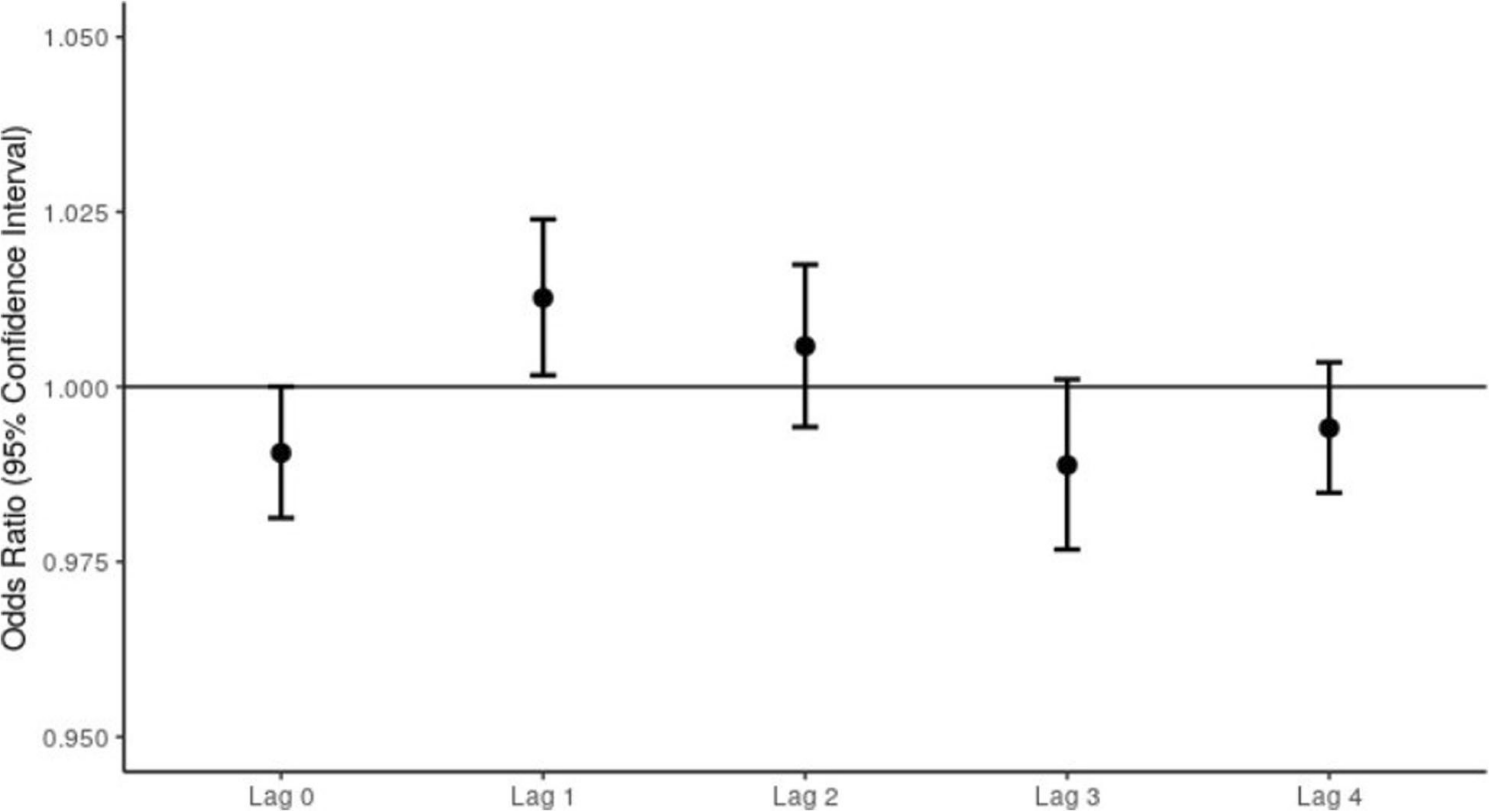
ORs of non-traumatic mortality on wildfire smoke days compared to non-wildfire smoke days from an unconstrained distributed lag model by lag day, adjusted by humidex

In Fig. 1, we display the results of our unconstrained distributed lag analysis, examining the effect of exposure to wildfire smoke in the 4 days prior to death. The pattern suggests that previous day exposure conveys the highest risk and that it diminishes rapidly such that there is no evidence of increased risk after 2 days. We estimate a 1.3% (95% CI: 0.2–2.4%) increase in the odds of non-traumatic mortality given wildfire smoke exposure on the previous day, while holding constant the humidex and exposure on lag days 0 and 2–4 (Fig. 1). The results indicate some evidence for an effect of exposure at 2 days prior to death, with inconclusive evidence for an effect of exposure in the preceding days on death.

In Table 3, we present the results of the inferential analysis, indicating a 1.0% (95% CI: − 1.0 – 4.0%) increase in the odds of all same-day non-traumatic mortality on wildfire smoke days compared to non-wildfire smoke days, controlling for humidex. We further observed a 1.0% (95% CI: − 6.0 – 4.0%) decrease in the odds of same-day cardiovascular mortality, a 9.0% (95% CI: 0.0–18.0%) increase in the odds of same-day respiratory mortality, and a 14.0% (95% CI: 2.0–26.0%) increase in the odds of same-day COPD mortality. Among ages 65–84, we observed a 2.0% (95% CI: − 2.0 – 6.0%) increase in the odds of all same-day non-traumatic mortality. With previous day exposure, we observed a 2.0% (95% CI, 0.0–5.0%) increase in the odds of all non-traumatic mortality, and a 5.0% (95% CI: − 3.0 – 15.0%) increase in the odds of respiratory mortality.

**Table 3:** Odds ratios (ORs) and 95% confidence intervals (CIs) for non-traumatic mortality for lag day 0 and lag day 1

Category	Lag Day 0: Adjusted OR (95% CI)	Lag Day 1: Adjusted OR (95% CI)	N (%) with exposure contrast^a^
All non-traumatic	1.01 (0.99, 1.04)	1.02 (1.00, 1.05)	31,719 (100)
Underlying cause of death			
Cardiovascular	0.99 (0.94, 1.04)	1.02 (0.97, 1.07)	8135 (25.6)
Ischemic heart disease	1.04 (0.93, 1.17)	1.00 (0.89, 1.13)	1482 (4.7)
Respiratory	1.09 (1.00, 1.18)	1.05 (0.97, 1.15)	2945 (9.3)
Asthma	0.51 (0.23, 1.12)	0.65 (0.31, 1.35)	46 (0.1)
COPD	1.14 (1.02, 1.26)*	1.07 (0.96, 1.20)	1732 (5.5)
Pneumonia	1.08 (0.86, 1.36)	0.97 (0.77, 1.22)	380 (1.2)
Cerebrovascular	0.90 (0.75, 1.07)	0.88 (0.74, 1.05)	710 (2.2)
Age group (years)			
0–4	0.97 (0.77, 1.22)	0.90 (0.71, 1.13)	423 (1.3)
5–14	0.95 (0.64, 1.41)	0.83 (0.54, 1.27)	140 (0.4)
15–44	0.99 (0.85, 1.15)	0.98 (0.85, 1.14)	935 (2.9)
45–64	1.00 (0.95, 1.06)	1.01 (0.95, 1.07)	6082 (19.2)
65–84	1.02 (0.98, 1.06)	1.03 (0.99, 1.07)	13,723 (43.3)
85+	1.00 (0.96, 1.05)	1.03 (0.98, 1.08)	10,416 (32.8)
Location			
Non-urban	1.00 (0.96, 1.03)	1.01 (0.97, 1.05)	17,770 (56.0)
Urban	1.02 (0.99,1.06)	1.03 (1.00, 1.07)	13,949 (44.0)
Median household income^b^			
< $35,000	0.99 (0.92, 1.07)	0.99 (0.92, 1.07)	3668 (11.6)
$35,000 - $50,000	1.05 (0.99, 1.10)	1.07 (1.02, 1.13)*	7921 (25.0)
$50,000 - $75,000	1.00 (0.96, 1.04)	1.00 (0.96, 1.05)	11,037 (34.8)
$75,000 - $100,000	1.03 (0.96, 1.10)	1.07 (1.00, 1.15)*	4579 (14.4)
≥ $100,000	0.98 (0.87, 1,11)	0.90 (0.80, 1.02)	1443 (4.5)
Race			
White	1.01 (0.99, 1.04)	1.03 (1.00, 1.06)*	28,395 (89.5)
Black	1.04 (0.89, 1.21)	0.99 (0.85, 1.16)	885 (2.8)
Native American	0.95 (0.77, 1.17)	0.79 (0.64, 0.98)*	472 (1.5)
Hispanic	0.81 (0.67, 0.99)*	0.91 (0.76, 1.10)	580 (1.8)
Native Hawaiian/ Other Pacific Islander	1.19 (0.93, 1.52)	1.04 (0.80, 1.34)	329 (1.0)
Asian	0.98 (0.84, 1.14)	0.99 (0.85, 1.15)	943 (3.0)
Sex			
Female	1.00 (0.96, 1.03)	1.03 (0.99, 1.06)	15,946 (50.3)
Male	1.03 (0.99, 1.06)	1.02 (0.98, 1.05)	15,772 (49.7)

*p ≤ 0.05

^a^Percent of cases, out of all cases whose strata have both wildfire smoke days and non-wildfire smoke days

^b^Annual median household income estimates at the census tract level

We conducted additional same-day and previous-day analyses, stratifying by age group, location, census tract median household income, race, and sex (Table 3). The majority of these analyses indicate little evidence for an effect, with odds ratios near 1.0 and confidence intervals consistent with null effects. However, when stratifying by race category, we observed a 19.0% (95% CI: −33.0 – −1.0%) decrease in the odds of same-day non-traumatic mortality among Hispanics, on wildfire smoke days compared to non-wildfire smoke days. Stratifying by census tract median household income, we observed a 7.0% (95% CI: 2.0–13.0%) increase, and a 7.0% (95% CI: 0.0–15.0%) increase in the odds of non-traumatic mortality with previous day exposure among those living in a census tract with median household income of $35,000 - $50,000 and $75,000–$100,000, respectively. We also observed a 3.0% (95% CI: 0.0–6.0) increase and a 21.0% (95% CI: −36.0 – −2.0%) decrease in non-traumatic mortality with previous day exposure among Whites and Native Americans, respectively. It is important to note, however, that many of the stratified groups have small sample sizes, limiting the power to detect an effect. Thus, the effect estimates should be interpreted with an appropriate degree of caution.

We conducted secondary analyses stratified by age group and race category within respiratory causes of death with same-day exposure (Table 4). For ages 45–64, we observed a 35.0% (95% CI: 9.0–67.0%) increase in the odds of same-day respiratory mortality, and a 33.0% (95% CI: 0.0–78.0%) increase in the odds of same-day COPD mortality. Within all respiratory mortality, we observed a 12.0% (95% CI: 2.0–22.0%) increase in the odds of same-day respiratory mortality among Whites.

**Table 4:** Odds ratios (ORs) and 95% confidence intervals (CIs) for stratification of same-day respiratory causes of death

Category	Adjusted OR (95% CI)	N (%) with exposure variation^a^
All respiratory causes of death		
Age group		
0–4	1.52 (0.58, 3.97)	22 (0.7)
5–14	–	–
15–44	0.91 (0.45, 1.84)	43 (1.5)
45–64	1.35 (1.09, 1.67)*	417 (14.2)
65–84	1.08 (0.96, 1.21)	1518 (51.5)
85+	1.00 (0.86, 1.16)	941 (32.0)
Race		
White	1.12 (1.02, 1.22)*	2697 (91.6)
Black	0.96 (0.53, 1.72)	62 (2.1)
Native American	0.88 (0.43, 1.82)	42 (1.4)
Hispanic	0.42 (0.19, 0.95)*	42 (1.4)
Native Hawaiian/ Other Pacific Islander	0.70 (0.20, 2.41)	16 (0.5)
Asian	0.88 (0.52, 1.53)	74 (2.5)
COPD causes of death		
Age group		
45–64	1.33 (1.00, 1.78)	238 (13.7)
65–84	1.14 (0.99, 1.31)	1002 (57.9)
85+	1.04 (0.85, 1.28)	481 (27.8)

* *p* ≤ 0.05

^a^Percent of cases, out of all cases whose strata have both wildfire smoke days and non-wildfire smoke days

## Discussion

Overall, our findings suggest evidence of a small increase in odds of all-ages non-traumatic mortality with same-day and with previous-day wildfire smoke exposure. Other mortality studies also provide evidence of an increase in the risk of non-accidental or natural mortality with same-day and previous day wildfire smoke exposure [18, 21, 23], including Johnston et al., that report a 1.0% (95% CI: −2.0 – 5.0%) and a 5.0% (95% CI: 0–10.0%) increase in the odds of non-accidental mortality with same-day and previous-day wildfire smoke exposure, respectively [21].

From our distributed lag model, we observed a 1.3% (95% CI: 0.0–2.0%) increase in the odds of next day all-ages, non-traumatic mortality for previous day wildfire smoke exposure versus non-wildfire smoke exposure, controlling for both humidex and exposure for the 4 days prior to death. Other studies report evidence for a lagged effect of non-traumatic mortality from wildfire smoke exposure from 1 to 5 days prior to death [18, 21], although few studies employ a distributed lag model [18]. Future wildfire smoke studies should employ distributed lag models, as is commonplace in air pollution epidemiology, to better capture the multiday lingering effects of exposure on health outcomes [42].

In examining specific causes of death, we observed estimates for all-ages cardiovascular mortality that are consistent with either an increase or decrease in risk, with same-day or previous-day wildfire smoke exposure. Other studies find evidence of an increased risk of all-ages cardiovascular mortality with both same-day and previous-day wildfire smoke exposure [21, 23]. However, Morgan et al. employed different exposure metrics, using PM_10_ rather than PM_2.5_ and reporting risk for each 10 μg/m^3^ increase in PM_10_, rather than comparing smoke days to non-smoke days [23]. Thus, we cannot easily compare risk estimates across studies.

Many of our subgroup analyses are limited by their more exploratory nature and wide confidence intervals, and thus must be interpreted with caution. However, we discuss two subgroup analyses that merit additional research to better understand the risk to each population.

In one subgroup analysis, we found evidence of an increase in the odds of all-ages COPD mortality with same-day and with previous-day wildfire smoke exposure. To our knowledge, no studies examine the association between wildfire smoke exposure and COPD mortality. However, populations with underlying health conditions, and in particular, asthma and COPD, have been found to be more susceptible to wildfire smoke compared to healthy populations in several studies examining hospital admissions and ED visits [23, 43–45]. Further research into the association between wildfire smoke exposure and COPD mortality is needed to confirm this observation.

In an additional subgroup analysis, we reported evidence of an increase in the odds of respiratory mortality among individuals ages 45–64 with same-day exposure. Several papers find higher effects of wildfire smoke exposure among adults under 65 compared to adults over 65, albeit for different health endpoints, none examining mortality as an endpoint for any age group [45–48]. Henderson et al. report the largest ORs for respiratory physician visits among adults ages 30–40 year [46], while Mott et al. find asthma and COPD hospital admissions greatest among adults ages 40–64 [47]. Rappold et al. find higher increases in respiratory ED visits (including asthma, COPD, pneumonia, and acute bronchitis) among individuals under 65 compared to those over 65 [48], and Reid et al. report higher COPD ED visits associated with PM_2.5_ in those ages 20–64 compared to over 65 [45]. However, most of the effect estimates we and others report are from secondary subgroup analyses with wide confidence intervals, meriting cautious interpretation. Nonetheless, people 45–64 with COPD are less likely to be using oxygen than people 65 and over with COPD, and thus more likely to be mobile and exposed to wildfire smoke [49]. Our findings suggest that underlying respiratory health conditions may contribute to the increased risk of respiratory and COPD-related mortality in the 45–64 age group. Thus, we recommend risk messaging target those of all ages with underlying health conditions, and specifically respiratory and COPD health conditions. Additional research is needed to further examine the risk to this population.

This analysis is limited by the challenges of separating anthropogenic PM_2.5_ and wildfire smoke PM_2.5_. We put considerable effort into determining a viable threshold between wildfire smoke affected PM_2.5_ and non-wildfire smoke affected PM_2.5_, but it is likely some misclassification exists [50]. We conducted a sensitivity analysis using 20.4 μg/m^3^ as the wildfire smoke-affected day threshold (see Additional file 1: Table S1), which showed the effects from wildfire smoke are sensitive to the exposure definition, and that the effect seen in this study may be capturing both the risk due to anthropogenic PM_2.5_ and wildfire smoke PM_2.5_. Further, existing wildfire smoke and mortality studies employ a wide variety of exposure assessment methods, which impedes direct comparison of effect estimates across studies. Additional research is needed to develop an ideal method to identify wildfire smoke-affected time periods.

Additional study limitations include the assumption that PM_2.5_ concentrations at each monitor represent the exposure for the population attributed to each monitor. Further, our method of assigning exposure allows individuals to be assigned to a different monitor on each day if a monitor is not working or is malfunctioning. This method, while not optimal, recoups data that would otherwise be dropped. Additionally, the air quality monitors do not represent the true exposure experienced by each person in the monitor grid cell area, likely resulting in some degree of exposure misclassification, a common limitation in air pollution epidemiology studies [51]. Further, exposures assigned from ambient PM_2.5_ concentrations do not reflect the reality that most people spend about 90% of their lives indoors [52], and additionally do not account for the steps people take to reduce exposures, where the main public health guidance during smoke episodes is to go indoors and keep indoor air clean [53].

Another limitation is that Washington State geo-coded mortality data may not correspond to the location of exposure for some decedents. We linked the geo-coded location of each decedent’s home residence to the corresponding grid cell. If the location of residence was not the location of exposure, this may result in some misclassification of exposure.

A final limitation of our study is regarding median household income, where an ecological indicator was employed, assigning the census tract median household income to each decedent. This method misclassifies individual income, but serves as a proxy for neighborhood-level socioeconomic status. Due to inadequate median household income data for 2006–2009, estimates for that time period are prone to higher rates of misclassification. Further analyses should develop more accurate proxies for individual or neighborhood-level socioeconomic status.

## Conclusion

This study is the first to estimate mortality risk associated with wildfire smoke exposure in Washington State. This study uses a tiered approach to exposure assessment, minimizing false allocation of anthropogenic PM-dominated days in urban areas as wildfire smoke days, as well as false allocation of smoke-dominated days in rural areas as anthropogenic PM-dominated days. This work will support local and state risk communication efforts and decision-making during future wildfire smoke events, especially for susceptible subpopulations identified in this study: those of all ages with COPD and other underlying respiratory conditions. Additional research is needed in Washington State to characterize the association between wildfire smoke exposure and less severe health endpoints of interest, including hospitalizations and ED visits, the health effects among vulnerable populations, as well as the health effects of prolonged smoke exposure.

## Supplementary information

**Additional file 1:** **Figure S1.** Number of cases of non-traumatic mortality at each stage of the study. **Figure S2.** Locations of the 75 regulatory air quality monitors in Washington State. **Text S1.** Exposure grid methods. **Figure S3.** Number of years with monitored exposure data for each 4 × 4 km grid cell. **Text S2.** Classification of monitors. **Table S1.** ORs and 95% CIs for all-ages, all same-day non-traumatic mortality associated with a > 20.4 μg/m^3^ threshold defining wildfire smoke days.

## Data Availability

The mortality data that support these findings are publicly available for a fee from the Washington State Department of Health. The air quality data that support these findings are publicly available from the Air Quality Program at the Washington State Department of Ecology upon request.

## Abbreviations

AIRPACT: Air Indicator Report for Public Awareness and Community Tracking model
CI: Confidence interval
COPD: Chronic Obstructive Pulmonary Disease
ED: Emergency Department
ICD: International Classification of Diseases
OR: Odds ratio
PM_2.5_: Particulate matter less than 2.5 μm in diameter
SD: Standard deviation
