# Supplementary material for: Mortality associated with wildfire smoke exposure in Washington state, 2006–2017: a case-crossover study
Source: Environ Health. 2020 Jan 13;19:4. doi: 10.1186/s12940-020-0559-2 (PMC6958692; doi:10.1186/s12940-020-0559-2)
Supplement: Supplementary file 1 — Additional file 1: Figure S1. Number of cases of non-traumatic mortality at each stage of the study. Figure S2. Locations of the 75 regulatory air quality monitors in Washington State. Text S1. Exposure grid methods. Figure S3. Number of years with monitored exposure data for each 4 × 4 km grid cell. Text S2. Classification of monitors. Table S1. ORs and 95% CIs for all-ages, all same-day non-traumatic mortality associated with a > 20.4 μg/m3 threshold defining wildfire smoke days. [file 12940_2020_559_MOESM1_ESM.docx]

**Additional file 1**

**Figure S1.** Number of cases of non-traumatic mortality at each stage of the study


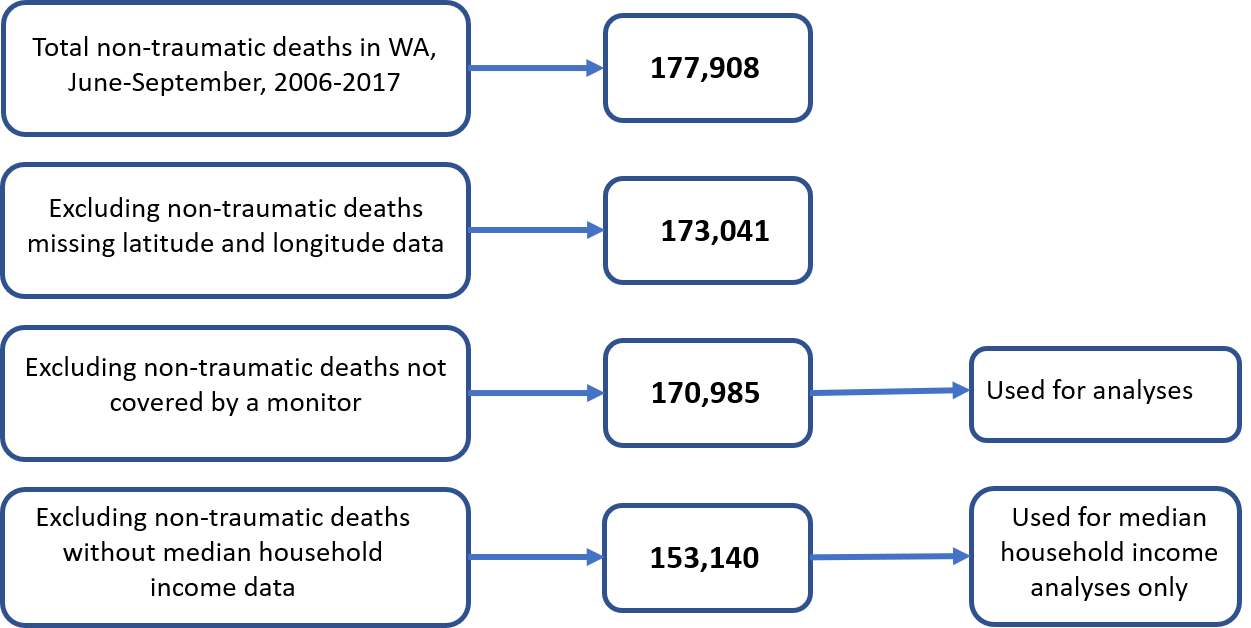


**Figure S2.** Locations of the 75 regulatory air quality monitors in Washington State


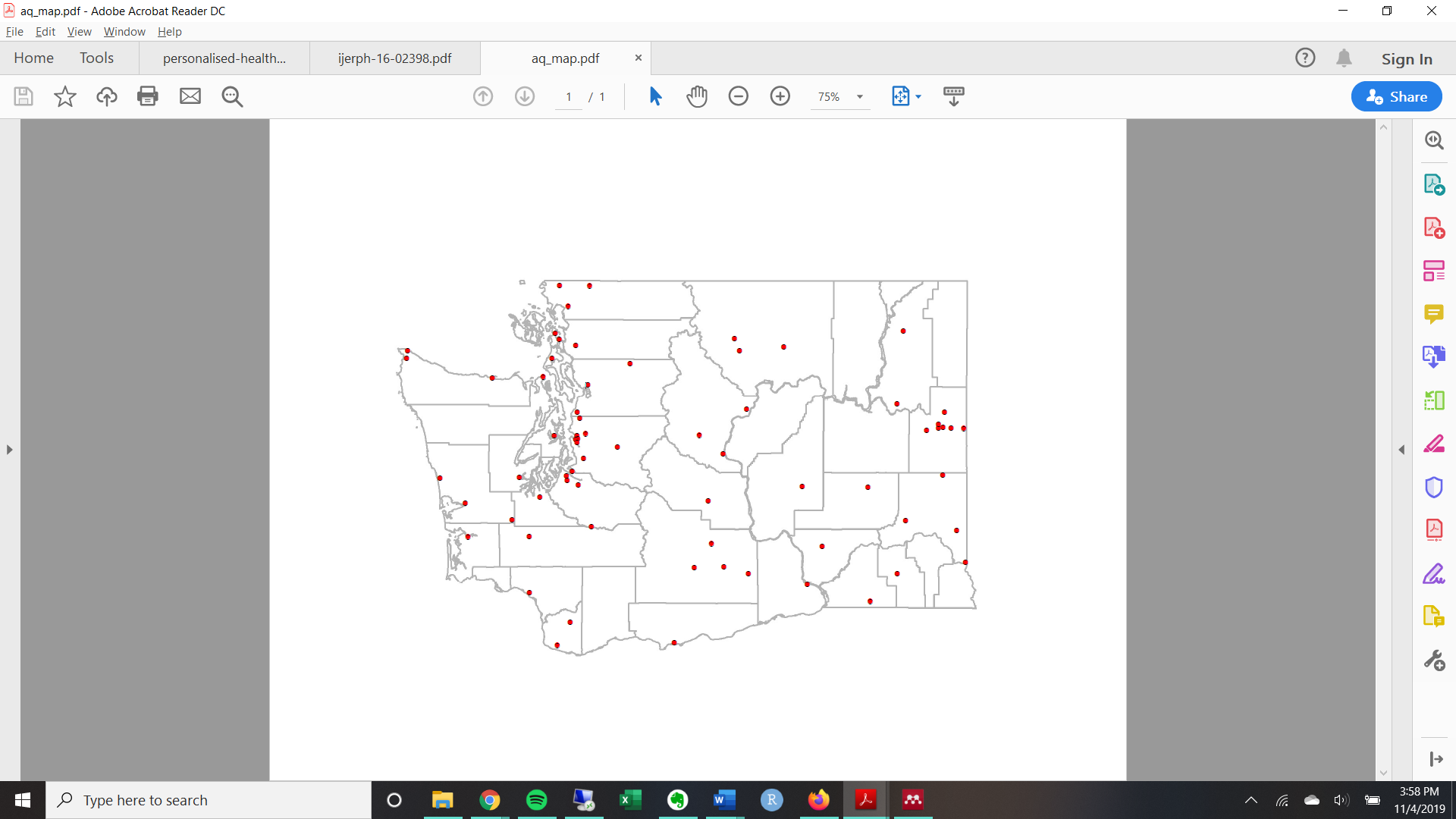


Text S1. Exposure grid methods

Air quality in Washington State is monitored at 75 State and Local Air Monitoring Stations (SLAMS) that measure one or more pollutants, including PM_2.5_, NO_2_, and CO, and meteorological variables, measured at 18 stations, including temperature, dew point, and wind speed (1). The spatial coverage of the 75 regulatory air quality monitors in Washington is not adequate for estimating daily exposure to wildfire smoke due to the unique terrain and meteorology (2). Instead, we relied on the *Air Indicator Report for Public Awareness and Community Tracking (AIRPACT-4)* modeled PM_2.5_, combined with the monitored daily PM_2.5_ concentrations to produce a more accurate exposure grid (3). The AIRPACT-4 model domain is composed of 4x4 km grid cells. AIRPACT-4 models air quality at high resolution across the Pacific Northwest. However, the AIRPACT-4 model does not produce reliable daily PM_2.5_ estimates, but rather, performs better over longer time periods. Further, AIRPACT-4 accurately predicts relative concentrations, that is, whether one area has a higher PM_2.5_ concentration than another area, but does a relatively poor job of predicting absolute concentrations. Thus, we decided to incorporate monitored PM_2.5_ concentrations into our exposure assessment.

To produce the PM_2.5_ gridded exposure data, we assigned empirical data from the 75 regulatory air quality monitors in Washington to corresponding area grid cells of the AIRPACT-4 model (3). Each monitoring site was assigned the ratio of its measured summer mean PM_2.5_ concentration to its AIRPACT-modeled summer mean PM_2.5_ concentration. These ratios were interpolated across the state using Empirical Bayesian Kriging (4). The interpolated ratio at each 4x4 km grid cell was then multiplied by the modeled 2014-2017 summertime mean PM_2.5_ to yield estimated summertime means across the state, at a 4x4 km resolution. Methods of merging modeled and monitored concentrations have been used in other instances, most notable in EPA’s Environmental Benefits Mapping and Analysis Program (BenMAP) (5,6). Many of the other examples of using the monitored and modeled concentrations estimate longer-term air quality concentrations, such as annual means. However, we needed 24-hour average PM_2.5_ concentrations, so using the monitored values was the best way to minimize misclassification. Finally, each air quality monitoring site was matched to the AIRPACT-4 model grid cell closest to it and to its nearest National Weather Service meteorological site in order to obtain the remaining meteorological variables. We used daily average temperature and dew point to calculate humidex, a measure of apparent temperature calculated from air temperature and dew point (7), for each monitor.

Next, the 4x4 km PM_2.5_ grid was overlaid with the Washington State Office of Financial Management’s (OFM) yearly population estimates at the census block group level (8). In cases where census block group boundaries cross grid cells, we determined the percent of the population’s census block group attributed to each grid cell by the area that fell within the grid cell. This method assumes populations are evenly distributed within each census block group and each grid cell. Grid cells were assigned the PM_2.5_ concentration of the nearest active representative monitor on each day. Monitors were considered representative of each grid cell if the difference between the summertime mean of the grid cell (determined above) and the nearest monitor was less than 2 µg/m^3^. If its nearest monitor was not representative or not active on a given day, the grid cell was assigned its second- or third-nearest monitor if both active and representative. Grid cells were then assigned the humidex value associated with their assigned PM_2.5_ monitor on each day. The end result is a dataset with the following for each day and each grid cell: 24-hour average PM_2.5_ concentration and humidex from a neighboring monitoring site with the population attributed to that grid cell. A small percentage of the grid cells and corresponding populations were not considered to be represented by any monitoring site, and were excluded from the analysis. This dataset was then joined with the above described mortality data. using a spatial join in ArcGIS (version 10.5.1) (Esri, Redlands, CA), assigning each latitude and longitude of death to the nearest grid cell and corresponding PM_2.5_ concentration and unit of humidex.

**Figure S3.** Number of years with monitored exposure data for each 4x4 km grid cell


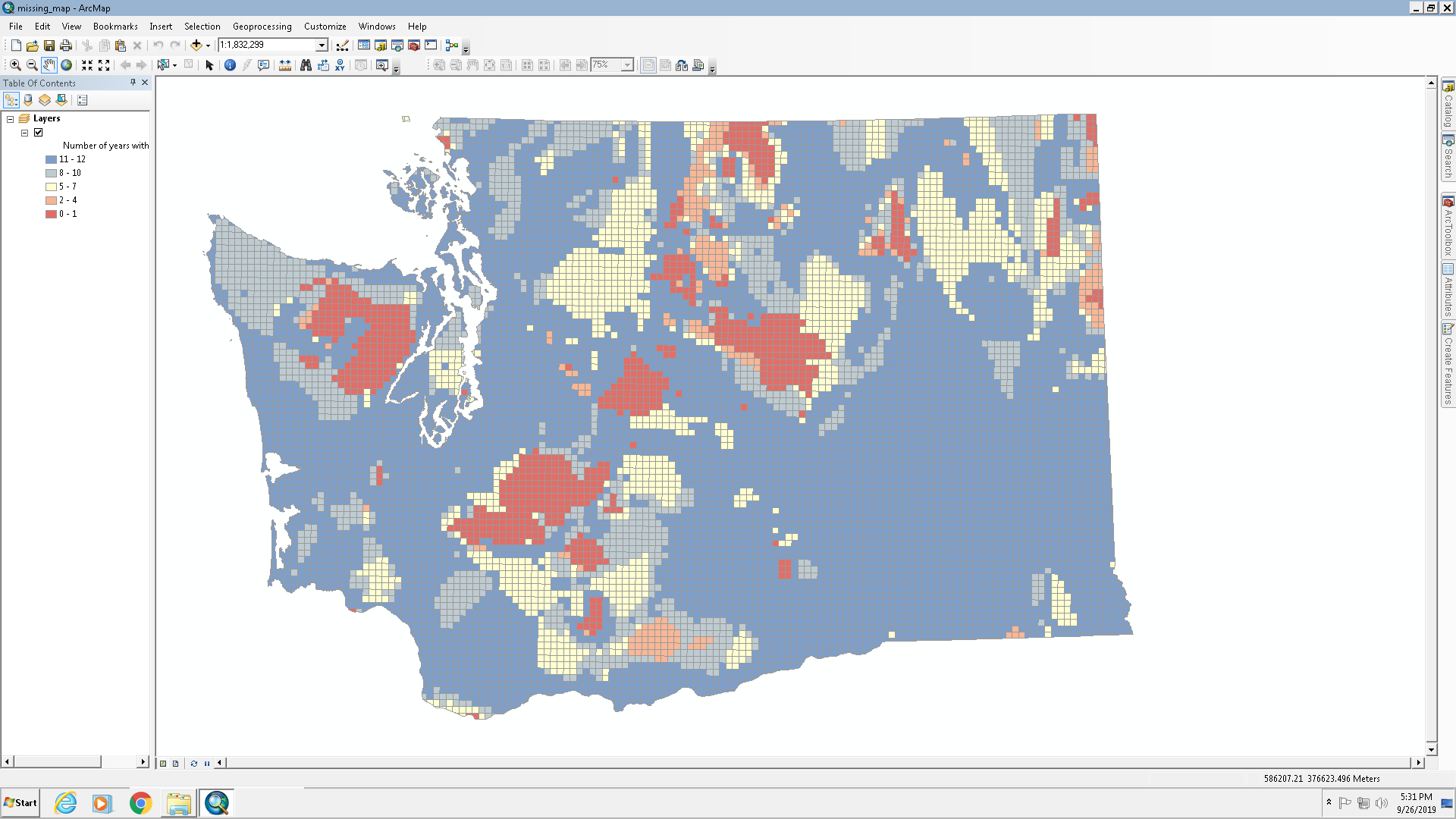


**Years with exposure data**

Text S2. Classification of monitors

The Washington Air Monitoring Network is made up of 75 air quality monitors capturing a variety of pollutants, with 59 monitoring fine particulate matter (PM_2.5_). In addition, local air agencies run additional monitoring sites outside the Washington Network; those sites are noted “non-network” below. In our determination of wildfire smoke days versus non-wildfire smoke day, half of the monitors in each of three urban areas had to be above 9 μg/m3 to be considered a wildfire smoke day. The three urban areas are Seattle, Tacoma, and Spokane. A list of the monitors in each urban area or cluster, is included below. All other monitors in the network were considered to be not urban for the purposes of this analysis.

Seattle: Lake Forest Park (AQS ID: 530330024); Bellevue (AQS ID: 530330031); Seattle-Duwamish (AQS ID: 530330057); Seattle-South Park (AQS ID: 530331011); Kent (AQS ID: 530332004); Lynnwood (AQS ID: 530610005); Seattle-10th and Weller (AQS ID: 530330030); Seattle-Beacon Hill (AQS ID: 530330080)

Tacoma: Tacoma-S 36th (AQS ID: 530530024); Tacoma-L (AQS ID: 530530029); Tacoma-Alexander (AQS ID: 530530031); Puyallup (AQS ID: 530531018)

Spokane: Spokane-Broadway (non-network); Spokane-Colbert-Greenbluff (non-network); Spokane-Monroe (AQS ID: 530630047); Spokane-Augusta (AQS ID: 530630021); Spokane-College (non-network); Airway Heights (non-network); Liberty Lake (non-network)

**Table S1.** ORs and 95% CIs for all-ages, all same-day non-traumatic mortality associated with a >20.4 μg/m^3^ PM_2.5_ threshold defining wildfire smoke days

| **Category** | **Adjusted OR (95% CI)** | **N (%) with exposure contrast^1^** |
| --- | --- | --- |
| All non-traumatic | 1.00 (0.96, 1.04) | 31,719 (100) |
| **Age group** |  |  |
| 0-4 | 0.94 (0.65, 1.38) | 423 (1.3) |
| 5-14 | 0.76 (0.38, 1.49) | 140 (0.4) |
| 15-44 | 0.83 (0.64, 1.06) | 935 (2.9) |
| 45-64 | 0.96 (0.87, 1.05) | 6,082 (19.2) |
| 65-84 | 1.02 (0.96, 1.08) | 13,723 (43.3) |
| 85+ | 1.04 (0.97, 1.12) | 10,416 (32.8) |
| **Underlying cause of death** |  |  |
| Cardiovascular | 0.96 (0.88, 1.03) | 8,135 (25.6) |
| Ischemic heart disease | 0.99 (0.82, 1.19) | 1,482 (4.7) |
|  |  |  |
| Respiratory | 1.10 (0.97, 1.24) | 2,945 (9.3) |
| Asthma | 0.56 (0.19, 1.71) | 46 (0.1) |
| COPD | 1.09 (0.93, 1.28) | 1,732 (5.5) |
| Pneumonia | 1.07 (0.73, 1.57) | 380 (1.2) |
|  |  |  |
| Cerebrovascular | 0.86 (0.66, 1.12) | 710 (2.2) |
| **Location** |  |  |
| Urban | 1.01 (0.96, 1.06) | 13,949 (44.0) |
| Non-urban | 0.99 (0.93, 1.05) | 17,770 (56.0) |
| **Race** |  |  |
| White | 1.00 (0.96, 1.04) | 28,395 (89.5) |
| Black | 0.99 (0.77, 1.27) | 885 (2.8) |
| Native American | 1.03 (0.76, 1.39) | 472 (1.5) |
| Hispanic | 0.74 (0.55, 1.00) | 580 (1.8) |
| Native Hawaiian/ Other Pacific Islander | 1.26 (0.88, 1.81) | 329 (1.0) |
| Asian | 1.18 (0.95, 1.47) | 943 (3.0) |
| **Median household income** |  |  |
| <$35,000 | 0.93 (0.82, 1.05) | 3,668 (11.6) |
| $35,000 - $50,000 | 1.02 (0.94, 1.10) | 7,921 (25.0) |
| $50,000 - $75,000 | 1.05 (0.99, 1.12) | 11,037 (34.8) |
| $75,000 - $100,000 | 0.99 (0.90, 1.10) | 4,579 (14.4) |
| ≥$100,000 | 0.96 (0.81, 1.15) | 1,443 (4.5) |

^1^Percent of cases whose strata have both wildfire smoke days and non-wildfire smoke days

**References**

1. Schulte J. 2018 Ambient Air Monitoring Network Plan. 2018.

2. Lassman W, Ford B, Gan RW, Fischer E V, Pierce JR. Spatial and temporal estimates of population exposure to wildfire smoke during the Washington state 2012 wildfire season using blended model, satellite, and in situ data. GeoHealth. 2017;1:106–21.

3. Washington State University. AIRPACT [Internet]. 2019. Available from: http://lar.wsu.edu/airpact/index.html

4. Pilz J, Spöck G. Why do we need and how should we implement Bayesian kriging methods. Stoch Environ Res Risk Assess. 2008;22(5):621–32.

5. Dhammapala R, Bowman C, Schulte J. A Monte Carlo method for summing modeled and background pollutant concentrations. J Air Waste Manage Assoc [Internet]. 2017;67(8):836–46. Available from: https://doi.org/10.1080/10962247.2017.1294546

6. Fann N, Lamson AD, Anenberg SC, Wesson K, Risley D, Hubbell BJ. Estimating the National Public Health Burden Associated with Exposure to Ambient PM 2 . 5 and Ozone. 2012;32(1).

7. Masterton J, Richardson F. Humidex: a method of quantifying human discomfort due to excessive heat and humidity. Downsview, Ontario: Environment Canada, Atmospheric Environment; 1979.

8. Management O of F. Population & demographics [Internet]. 2017. Available from: https://www.ofm.wa.gov/washington-data-research/population-demographics
